# Phenotypic characteristics of early Wolfram syndrome

**DOI:** 10.1186/1750-1172-8-64

**Published:** 2013-04-27

**Authors:** Bess A Marshall, M Alan Permutt, Alexander R Paciorkowski, James Hoekel, Roanne Karzon, Jon Wasson, Amy Viehover, Neil H White, Joshua S Shimony, Linda Manwaring, Paul Austin, Timothy E Hullar, Tamara Hershey

**Affiliations:** 1 Department of Pediatrics, Washington University in St. Louis School of Medicine, St. Louis, MO 63110, USA; 2 Department of Internal Medicine, Washington University in St. Louis School of Medicine, St. Louis, MO 63110, USA; 3 Department of Neurology, Center for Neural Development and Disease, University of Rochester Medical Center, Rochester, NY, USA; 4 Department of Pediatrics, Center for Neural Development and Disease, University of Rochester Medical Center, Rochester, NY, USA; 5 Department of Biomedical Genetics, Center for Neural Development and Disease, University of Rochester Medical Center, Rochester, NY, USA; 6 Department of Ophthalmology, Washington University in St. Louis School of Medicine, St. Louis, MO 63110, USA; 7 Department of Otolaryngology-Head and Neck Surgery, Washington University in St. Louis School of Medicine, St. Louis, MO 63110, USA; 8 Department of Audiology, Washington University in St. Louis School of Medicine, St. Louis, MO 63110, USA; 9 Department of Neurology, Washington University in St. Louis School of Medicine, St. Louis, MO 63110, USA; 10 Department of Mallinckrodt Institute of Radiology, Washington University in St. Louis School of Medicine, St. Louis, MO 63110, USA; 11 Department of Surgery, Washington University in St. Louis School of Medicine, St. Louis, MO 63110, USA; 12 Department of Urology, Washington University in St. Louis School of Medicine, St. Louis, MO 63110, USA; 13 Department of Psychiatry, Washington University in St. Louis School of Medicine, St. Louis, MO 63110, USA; 14 St. Louis Children’s Hospital, One Children’s Place, St. Louis, MO 63110, USA; 15 Department of Pediatrics, Division of Endocrinology and Diabetes, One Children’s Place, Campus Box 8116, St. Louis, MO 63110, USA

**Keywords:** Diabetes mellitus, DIDMOAD, Diabetes insipidus, Hearing loss, Optic atrophy, Color blindness, Neurodegenerative disorder

## Abstract

**Background:**

Wolfram Syndrome (WFS:OMIM 222300) is an autosomal recessive, progressive, neurologic and endocrinologic degenerative disorder caused by mutations in the WFS1 gene, encoding the endoplasmic reticulum (ER) protein wolframin, thought to be involved in the regulation of ER stress. This paper reports a cross section of data from the Washington University WFS Research Clinic, a longitudinal study to collect detailed phenotypic data on a group of young subjects in preparation for studies of therapeutic interventions.

**Methods:**

Eighteen subjects (ages 5.9–25.8, mean 14.2 years) with genetically confirmed WFS were identified through the Washington University International Wolfram Registry. Examinations included: general medical, neurologic, ophthalmologic, audiologic, vestibular, and urologic exams, cognitive testing and neuroimaging.

**Results:**

Seventeen (94%) had diabetes mellitus with the average age of diabetes onset of 6.3 ± 3.5 years. Diabetes insipidus was diagnosed in 13 (72%) at an average age of 10.6 ± 3.3 years. Seventeen (94%) had optic disc pallor and defects in color vision, 14 (78%) had hearing loss and 13 (72%) had olfactory defects, eight (44%) had impaired vibration sensation. Enuresis was reported by four (22%) and nocturia by three (17%). Of the 11 tested for bladder emptying, five (45%) had elevated post-void residual bladder volume.

**Conclusions:**

WFS causes multiple endocrine and neurologic deficits detectable on exam, even early in the course of the disease. Defects in olfaction have been underappreciated. The proposed mechanism of these deficits in WFS is ER stress-induced damage to neuronal and hormone-producing cells. This group of subjects with detailed clinical phenotyping provides a pool for testing proposed treatments for ER stress. Longitudinal follow-up is necessary for establishing the natural history and identifying potential biomarkers of progression.

## Background

Wolfram syndrome (WFS) (OMIM #222300) is a rare autosomal recessive genetic disease characterized by insulin dependent diabetes mellitus, optic atrophy, blindness, hearing loss, and other neurological dysfunctions resulting in death from widespread neurodegeneration in the third or fourth decade [1]. The causative gene (WFS1) [2], which encodes the wolframin protein, has been identified, and a number of loss-of-function mutations have been described [2,3]. Cell [4] and animal models [5] have led to the discovery that the wolframin protein resides in the endoplasmic reticulum (ER) membrane [6], and that mutant forms predispose insulin-producing pancreatic β-cells to ER stress-mediated apoptosis [7-9]. Both type 1 and type 2 diabetes have been shown to result from a variety of genetic risk factors and environmental components. These factors impinge on pancreatic β-cells resulting in ER stress, oxidative stress, impaired signal transduction, and mitochondrial dysfunction, all combining to result in β-cell apoptosis and diabetes[10-13]. The complexity of type 1 and type 2 diabetes makes it difficult to discern the contribution of any one gene or pathogenic mechanism. In contrast to the multifactorial etiology of diabetes, subjects with WFS represent an early-onset form of diabetes and neurodegeneration due to deficiency of a single protein, making WFS an ideal model of an ER stress disorder. Currently, no intervention is known to alter the progression or life expectancy in WFS, but earlier recognition of the syndrome in individuals could improve their quality of life by allowing earlier intervention for the various debilitating components of WFS.

While there are numerous reports of the features of WFS from retrospective records [1,3,14-18], there are no direct, detailed clinical observations of a young cohort studied in one center by the same observers. This study represents a cross-sectional analysis of a group of subjects relatively early in the disease with varying duration and manifestations of the syndrome. The ultimate objective of this study is to observe subjects with WFS longitudinally beginning early in the disease and to identify appropriate biomarkers for monitoring disease progression, thus preparing for future clinical trials. We report the detailed clinical phenotype of a group of WFS subjects seen at our annual multidisciplinary Wolfram Syndrome Research Clinic at Washington University,

## Methods

### Subjects

Eighteen participants, 3 of whom were siblings, aged 5 to 25 were recruited through the Washington University Wolfram Syndrome International Registry website (http://wolframsyndrome.dom.wustl.edu/medical-research/Wolfram-Syndrome-Home.aspx) to participate in standardized evaluations across domains known to be associated with WFS. In most cases, subjects initiated joining the registry and so were self-selected and the investigators further selected those who were young and in the early stages of the syndrome to take part in the research clinic. Inclusion criteria for the registry are the diagnosis of insulin-dependent diabetes mellitus and optic atrophy by a physician before 18 years of age and/or genetic confirmation of a WFS1 mutation. Subjects are excluded from the registry if they or a guardian are unable to speak English or to obtain help with translation and thus are unable to understand the registry questionnaires. The clinic protocol was approved in advance by the Washington University Human Research Protection Office and all subjects provided informed consent if adults and assent with consent by parents if minor children. Subject’s identification numbers shown in this report are based on their numbers in the registry and are therefore not necessarily consecutive.

### Methods

#### Overview

All subjects attended the Washington University WFS Research Clinic in St. Louis, MO, in August 2010, July 2011, and/or July 2012. This report includes data from each subject’s initial evaluation. All subjects had a complete medical and family history and a physical examination (including a mixed meal challenge test) by a pediatric endocrinologist (BM or NHW); a neurologic history and complete neurologic exam by a pediatric neurologist (AP and AV); visual acuity, refraction, assessment of nystagmus, color vision testing, pupillary testing and dilated fundus exams by a pediatric optometrist (JH); audiology exams supervised by an audiologist (RK); complete family history by a genetic counselor (LM); vestibular examination by an neurotolologist (TEH); urologic evaluation by a pediatric urologist (PA) and neuroimaging supervised by a pediatric neuroradiologist (JS), and psychological, and cognitive testing supervised by a neuropsychologist (TH).

#### Specific tests

##### Mixed meal challenge tests

All subjects but one had diabetes mellitus and all diabetic subjects were taking insulin either by multiple daily injections (Lantus and a rapid-acting insulin analog) or by insulin pump. The night before the oral mixed meal tolerance test, the subjects turned their pump basal rate to 50% of the normal rate at midnight or took half of their evening dose of Lantus insulin and fasted from midnight until the test at 8 a.m. The mixed meal consisted of 6 ml/kg (maximum 360 ml) of Boost. Blood for glucose and C-peptide measurement was drawn at time 0 (fasting) and 30 minutes after the Boost. If a subject’s fasting glucose exceeded 11.1 mmol/l, the test was not performed, but fasting glucose and C-peptide were obtained.

##### Audiology and vestibular testing

Pure tone testing (250 to 8,000 Hz) was administered via insert earphones with a Madsen Orbiter 922 audiometer calibrated to American National Standards Institute [ANSI] S3.6-1996 [19]. WU.WOLF-05 and WU.WOLF-12 were not tested at the multidisciplinary clinic; audiologic data were obtained from their local audiologist. To test vestibular function, harmonic sinusoidal rotations about the earth-vertical axis were administered to 8 subjects over the frequency range of 0.025 to 0.5 Hz at a peak velocity of 60 deg/sec using a clinical rotational chair and video eye movement-recording device (Micromedical Technologies, Chatham, IL). All subjects were tested only with low-amplitude rotational stimulation; this is not sensitive to all vestibular losses.

##### Neuroimaging

MRI scans were acquired in the Center for Clinical Imaging Research on a Siemens 3 T Tim Trio Magnetic Resonance Imaging (MRI) scanner. Blood glucose was confirmed to be above 4 mmol/l prior to scanning. T1-weighted (sagittal acquisition, TR = 2,400, TE = 3.16, TI = 1,000, voxel res = 1 × 1 × 1 mm, frames = 176, Flip = 8, Time = 8:09) and T2 FLAIR images (transverse acquisition, TR = 9,190, TE = 98, TI = 2,500, voxel res = .86 × .86 × 3 mm, frames = 42, Flip = 150, Time = 3:59) were read by a pediatric neuroradiologist. The evaluation of the pituitary bright spot was performed qualitatively by a pediatric neuroradiologist with over 10 years of experience (JSS). The initial interpretation of the scans was done as they were acquired, however the final evaluation of the pituitary for all subjects was performed in one sitting in order to facilitate comparison across the group.

##### Cognitive testing

Blood glucose was confirmed to be above 4 mmol/l prior to testing.

The Wechsler Abbreviated Scale of Intelligence (WASI) [20] Vocabulary and Similarities subtasks were administered to allow us to generate estimated verbal intelligence quotients (IQ) by comparing to age norms.

##### University of Pennsylvania smell identification test (UPSIT) [21]

To test olfaction, we administered a scratch-and-sniff test of common odorants. The UPSIT has high reliability (test-retest r = 0.94) and established sensitivity [21]. The UPSIT defines degrees of olfactory sensitivity based on how many scents the subject identifies correctly. Subjects can be classified as having normosmia (for age 5–9 = 19 or more correct, for age 10–14 = 32 or more correct, for age 15 and up = 35 or more correct for female, 34 or more correct for male), mild microsmia (for age 10–14 = 28–31 correct, for age 15–29 = 31–34 correct for female, 30–33 correct for male), moderate microsmia (for age 10–14 = 23–27 correct, for age 15 and up = 26–30 correct for female, 26–29 correct for male), severe microsmia (for age 10–14 = 19–22 correct, for age 15 and up = 19–25 correct), total anosmia (6–18 correct), or “probable malingering” (fewer than 6 correct) based on published gender and age-adjusted norms [22].

## Results

### Clinical characteristics and mutation analysis

There were 18 subjects with WFS with an average age of 14.2 ± 5.7 years at their first visit to the research clinic. The ages of the seven male subjects ranged from 7.3 to 22.9 years (average of 13.7 ± 5.4) and the ages of the eleven female subjects from 5.9 to 25.8 years (average of 14.7 ± 5.8). All subjects had been diagnosed with both diabetes mellitus (DM) and optic atrophy prior to their initial clinic visit except one, who was diagnosed with WFS due to the presence of insulin requiring diabetes mellitus and a family history of the condition, and one who was diagnosed with WFS due to genetic testing done due to findings of optic atrophy and hearing loss. Table 1 shows the family and medical history for each subject.

**Table 1:** Subject medical and family history

**Subject**	**Age**	**Gender**	**DM onset**	**DI onset**	**Optic atrophy onset**	**Hearing loss**	**Diagnosed with WFS**	**FH WFS**	FH DM*	FH hearing loss*	**Other problems**
WU.WOLF-01	13.1	M	3.5	9	5	9	N/A	N	Father	N	GERD, constipation, headaches, restless legs, hypothyroidism, hypogonadism
WU.WOLF-02	10.9	F	6	7.5	9	N	9.5	N	Mgaunt	Pgf	None
WU.WOLF-03	17.9	M	5.0	6	6	6	6	N	Mgf	mgm	GERD; restless legs; occasional myoclonus
WU.WOLF-04	23.8	F	2.3	12	5	5	12	N	Father, pgm, paunts	Father	GERD; OCD spectrum behaviors; constipation, loss of taste sensation
WU.WOLF-05	13.8	F	3.8	N	12	1.7	13	N	Mgf, mggm, mgaunt	mcousin	FTT at 1 year, feeding tube at age 2 1/2; Near-drowning age 6 ½; celiac sprue
WU.WOLF-07	7.3	M	2.7	7	N	7	3	Y†	Mgf, paunt, puncle, mggf, mguncle	Pgf, 3 pguncles	anxiety
WU.WOLF-09	14.3	M	10.8	14	11	N	N/A	Y‡	Distant cousin, pggf	Maunt, muncle, pguncle, mggf	Asthma, migraines, obstructive sleep apnea constipation,
WU.WOLF-10	11.7	F	7.0	11	9	N	8	Y‡	Distant cousin, pggf	Maunt, muncle, pguncle, mggf	GERD, headaches, night terrors, constipation
WU.WOLF-11	8.3	M	7.5	10	6	8	7	Y‡	Distant cousin, pggf	Maunt, muncle, pguncle, mggf	headaches, constipation, loss of normal taste—“things taste funny”
WU.WOLF-12	22.9	M	7.0	17	7	7	17	N	Father (after kidney cancer), pgf, puncle, pggf, mgf, pguncle	N	GERD, myoclonus, neurogenic bladder, chronic renal failure, hypogonadism, loss of taste sensation
WU.WOLF-13	5.9	F	4.8	7.5	5.2	6	5.4	N	first cousin	N	Constipation
WU.WOLF-14	13.6	F	6.3	11.3	7.9	10.1	8.8	N	Mgf, pggm, mggf	N	None
WU.WOLF-15	10.8	F	2.8	N	7	9	7	N	N	brother	Constipation
WU.WOLF-16	25.8	F	13.1	14.6	13.6	25.8	14.9	N	pgf	pgm	None
WU.WOLF-17	17.2	F	5	N	15.3	16.3	16.6	N	Mother, maunt, mgm, mgf, mggf, mgaunt, mguncle, mcousin	mgaunt	None
WU.WOLF-18	11.9	M	5.1	10.33	10.1	11.9	10.6	N	Mgf	Father	None
WU.WOLF-19	11.9	F	N	N	5	Prior to 5	11	No info	No info	No info	International adoption age 5, maternal half sibling is healthy.
											Constipation
WU.WOLF-22	15.8	F	14	N	13	N	14.6	N	Mggf, pgm, pgf	N	None
**MEAN**	**14.2**		**6.3**	**10.6**	**8.7**	9.0	10.3				
**STD**	**5.7**		**3.5**	**3.3**	**3.3**	6.0	4.2				

*Not in a person with Wolfram syndrome.

†3 distant cousins with Wolfram, parents consanguinous.

‡Sibling with Wolfram (patients WU.WOLF-11, WU.WOLF-10,WU.WOLF-09 are siblings).

Abbreviations: DI: diabetes insipidus; DM: diabetes mellitus; FH: family history; FTT: failure to thrive; GERD: gastroesophageal reflux disease; maunt: maternal aunt; mcousin: maternal cousin; mgaunt: maternal great aunt; mgf: maternal grandfather; mggf: maternal great grandfather; mggm:maternal great grandmother; mgm: maternal grandmother; mguncle: maternal great uncle; muncle: maternal uncle; OCD: obsessive compulsive disorder; paunt: paternal aunt; pgaunt: paternal great aunt; pgf: paternal grandfather; pgm: paternal grandmother; pguncle: paternal great uncle; puncle: paternal uncle; WFS: Wolfram Syndrome.

The average age of onset of DM was 6.3 ± 3.5 years for the 17 (94%) who had DM. One female, WU.WOLF-19, had not yet developed DM at age 11.9 years. Of the remaining ten females, the average age of onset was 6.5 ± 4 years, but without a normal distribution: two were diagnosed in the third year of life, one each in the fourth, fifth, and sixth, two in the seventh, one in the eighth, then a skip to the 14^th^ and 15^th^ year of life. Of the male subjects, the average age of onset of DM was 5.9 ± 2.7, with a similar age distribution to the females except there were no males who did not have DM by age 10.8 years.

Turning to diabetes insipidus (DI), the average age of onset for the entire group was 10.6 ± 3.3 years, and 13 subjects (72%) had DI. All of the males had DI with an average age of onset of 10.5 ± 3.9 years and an age of onset range from 6 to 17 years. Six of the eleven female (55%) subjects had DI, with an average age of onset of 10.7 ± 2.8 years and a range of 7.5–14.6 years. Those female subjects who did not have DI had an average age of 13.9 ± 2.7 and a range of 10.8–15.8 years. This suggests that males may have a tendency toward an earlier onset of DI, though the number of subjects is small.

Optic atrophy was diagnosed in all but one subject (94%). The average age of diagnosis was 8.7 ± 3.3 years for all subjects with optic atrophy, 7.5 ± 2.4 (range 5–11) years for the males, and 9.3 ± 3.7 (range 5–15.3) years for the females.

Nine subjects (50%) had previously-diagnosed hearing loss and 5 more (for a total of 78%) were newly diagnosed with hearing loss at the clinic. Six of the seven male subjects had hearing loss with a mean age of diagnosis of 8.2 ± 2.1 (range 6–11.9) years. The one boy with no loss still had normal hearing at age 14.3 years. The mean age of diagnosis of hearing loss in the female subjects was 9.9 years but the group again did not exhibit a normal distribution. The standard deviation was 7.8 years for onset of hearing loss in the female group and three of the 11 females (27%) had no hearing loss at ages 10.9, 11.7, and 15.8 years. Of those with hearing loss, two had profound hearing loss from very early in life—subject WU.WOLF-05 diagnosed at age 1.7 years and subject WU.WOLF-19 who was profoundly deaf with no language development at the time of her adoption at age 5 (note—age 5 was used as her “official” age of hearing loss diagnosis in the table and calculation of the average age of onset of hearing loss as that is the youngest age that we can be certain that she had hearing loss). Although it is not possible to determine at what age she developed hearing loss, anecdotal evidence suggested that she never had functional hearing. There are two of the female subjects who developed hearing loss late, at ages 25.8 and 16.3 years, and the remaining group of four developed hearing loss at an average age of 7.5 ± 2.4 years.

The majority of subjects had a family history of diabetes or hearing loss in relatives who are not believed to have WFS, though none of the relatives with hearing loss has been tested for WFS as far as we are aware besides those noted to have WFS. Table 2 shows the mutations in the WFS1 gene that are present in each subject. Two subjects had mutations found in only one allele and one of those, c.937C > T; p.H313Y, has been reported to present in an autosomal dominant fashion in a subject who, like this one, presented with very early profound hearing loss [23].

**Table 2:** Mutation analysis of the WFS1 gene

**Subject**	**Allele 1**	**Allele 2**
WU.WOLF-01	c.1060-1062delTTC; p.F354del	c.2663C>A; p.S888X
WU.WOLF-02	fc.2648del4; p.F883fsX950	No mutation
WU.WOLF-03	c.1230-1233delCTCT; p.Val412fs440Stop	c.1243-1245delGTC: p.Val415del
WU.WOLF-04	c.1112G>A; p.W371X	c.1885C>T; p.R629W
WU.WOLF-05	c.937C>T; p.H313Y	No mutation
WU.WOLF-07	c.2002C>T; p.Q668X	c.2002C>T; p.Q668X
WU.WOLF-09	c.376G>A; p.A126T	c.1838G>A;p.W613X
WU.WOLF-10	c.376G>A; p.A126T	c.1838G>A;p.W613X
WU.WOLF-11	c.376G>A; p.A126T	c.1838G>A;p.W613X
WU.WOLF-12	c.320G>A; p.G107E	c.1882C>T; p.R629W
WU.WOLF-13	c.599delT; p.L200fs286Stop	c.2254G>T; pE752Stop
WU.WOLF-14	c.817G>T; p.E273X	c.1839G>A; p.W613X
WU.WOLF-15	c.439delC, p.R147fsX163	c.1620G>A, p.W540X
WU.WOLF-16	c.1240_1242delTTC; p.F414del	c.1689_1694delCTTCTT; p.F564del; p.L565del
WU.WOLF-17	c.599T>C; p.L200P	c.695G>C; p.R232P
WU.WOLF-18	c.1251_1252delCTinsG; p.Phe417Leufsx25	c.1885C>T; p.Arg629Trp
WU.WOLF-19	c.2339G>C, p.Gly780Ala	c.2452C>T, p.Arg818Cys
WU.WOLF-22	c.605A>G; p.E202G	c.631G>A; p.D211N

### History and review of systems

Only four (22%) of the subjects had a normal birth history, the others reporting meconium aspiration, preterm labor, breech presentation, or failure to progress in labor. All of the subjects had normal language development at the time of the clinic. A near drowning incident in early childhood in WU.WOLF-05 had residual neurologic effects. A history of provoked seizures (due to fever or hypoglycemia) was found in three (17%). Significant psychiatric issues were reported in two (11%), including repetitive behaviors, obsessive compulsive disorder, and auditory hallucinations. Constipation (seven subjects, 39%) and a loss of normal taste sensation (three subjects, 17%) were reported in several.

### Diabetes mellitus

The results of the mixed meal tolerance test are shown in Table 3. WU.WOLF-19 was not diabetic either by history, by hemoglobin A1c testing, or by glucose tolerance testing and she had a strong C-peptide response. Two subjects (11%) had a rise in C-peptide of greater than 0.3 ng/ml in response to the mixed meal and both of those had been diagnosed with diabetes within the past 2 years. A measureable rise in C-peptide or a C-peptide of 1.0 or higher at baseline was found in seven (39%) of the subjects. The mean hemoglobin A1c was 7.7%, indicating fairly good glycemic control. Only two (11%) of subjects reported a history of diabetic ketoacidosis and one (6%) reported a history of hypoglycemic seizures.

**Table 3:** Hemoglobin A1c and oral glucose tolerance test results

**Subject**	**HgbA1c**	**Fasting glucose**	**Fasting C-peptide**	**Glucose at time 30 min**	**C-peptide at time 30 min**
WU.WOLF-01	8.1	135	0.1	352	0.4
WU.WOLF-02	8	287	1.0	ND	ND
WU.WOLF-03	6.2	190	0.5	205	0.4
WU.WOLF-04	7.5	277	0.1	ND	ND
WU.WOLF-05	7.6	91	0.1	283	0.2
WU.WOLF-07	6.7	103	0.1	238	0.3
WU.WOLF-09	11.5	280	0.2	ND	ND
WU.WOLF-10	8	167	0.2	305	0.4
WU.WOLF-11	6.6	139	0.9	202	1.8
WU.WOLF-12	6.5	231	0.1	302	0.2
WU.WOLF-13	8.0	ND	ND	ND	ND
WU.WOLF-14	9.3	269	0.5	ND	ND
WU.WOLF-15	8.2	167	0.2	265	0.3
WU.WOLF-16	7.7	144	<0.1	194	0.2
WU.WOLF-17	9.3	257	0.2	ND	ND
WU.WOLF-18	8.3	267	0.2	ND	ND
WU.WOLF-19	5.1	85	2.1	113	9.3
WU.WOLF-22	6.5	184	0.9	266	2.6
**MEAN**	7.7	192.5	0.5	247.7	1.5
**STD**	1.4	70.9	0.5	66.4	2.7

ND = Not done.

### Neurologic and neuropsychological assessments

On exam, most subjects had some abnormal findings (Table 4), with ten (56%) showing gait abnormalities apparent on a standard detailed neurologic exam. However, on detailed specific testing for gait and balance disturbances, this group of WFS subjects was found to walk more slowly, take shorter and wider steps, and spend more time in double support than an age matched group of healthy control individuals (data previously published [24,25]). Vibration sensation was impaired in eight (44%). Other neurologic deficits included slow motor movements in three (17%) and increased tone and brisk deep tendon reflexes in one.

**Table 4:** Neurologic exam findings

**Subject**	**Motor**	**Deep_Tendon reflexes**	**Sensation**	**Coordination**	**Gait**
WU.WOLF-01	Increased tone lower extremities; intention tremor	Brisk 3+/4 at ankles	Normal	Mild dysmetria	Unable to tandem walk
WU.WOLF-02	Normal	Normal	Normal	Normal	Normal
WU.WOLF-03	Normal	Normal	Impaired vibration (R6/L4)	Normal	Abnormal tandem walk
WU.WOLF-04	Fine finger movements slow; toe tapping slow	Normal	Impaired vibration (R4/L2)	Mild dysmetria (probably due to visual impairment)	Wide-based gait; unable to tandem walk
WU.WOLF-05	toe tapping slow	Normal	Normal	Normal	Abnormal tandem walk
WU.WOLF-07	Normal	Normal	Normal	Normal	Normal
WU.WOLF-09	Normal	Normal	Impaired vibration (R4/L4)	Normal	Abnormal tandem walk
WU.WOLF-10	Normal	Normal	Normal	Normal	Normal
WU.WOLF-11	Normal	Normal	Normal	Normal	Abnormal tandem walk
WU.WOLF-12	Normal	Normal	Impaired vibration (R2/L2)	Normal	Abnormal tandem walk
WU.WOLF-13	Normal	Normal	Normal	Normal	Normal
WU.WOLF-14	Fine finger movements slow	Normal	Normal	Normal	Normal
WU.WOLF-15	Normal	Normal	Normal	Normal	Normal
WU.WOLF-16	Normal	Normal	Mildly impaired vibration	Mild dysmetria	Abnormal tandem walk
WU.WOLF-17	Normal	Normal	Mildly impaired vibration	Some difficulty hopping	Abnormal tandem walk
WU.WOLF-18	Normal	Normal	Mildly impaired vibration	Normal	Mildly abnormal tandem walk
WU.WOLF-19	Normal	Normal	Difficult to assess, probably normal	Normal	Normal
WU.WOLF-22	Normal	Normal	Mildly impaired vibration	Normal	Normal

Disorders of olfaction were common (13 subjects = 72%) (Table 5). Four (57%, average age 15.6 years) of the males has severe microsmia or total anosmia, while one had normal olfaction at age 7.3 and two others had mild to moderate defects (ages 14.3 and 11.9). Three (27%, average age 13.5 years) of the females had severe microsmia or total anosmia, while four (36%, average age 13.7 years) had normal olfaction and four (36%, average age 16.5 years) had mild or moderate microsmia.

**Table 5:** Olfaction, verbal IQ, neuroimaging, and audiology testing findings

**Subject**	**Test of olfaction**	**Verbal IQ range**	**Clinical neuroimaging findings**	**Hearing loss**	**Type of hearing loss**
WU.WOLF-01	Total Anosmia	ND	ND	Yes	Sensorineural
WU.WOLF-02	Mild Microsmia	Very Superior	Normal	No	Normal
WU.WOLF-03	Total Anosmia	High Average	No pituitary bright spot	Yes—hearing aid at age 6	Sensorineural
WU.WOLF-04	Severe Microsmia	Average	ND	Yes—hearing aid at age 12	Sensorineural
WU.WOLF-05	Normosmia	Borderline	ND	Yes—cochlear implant at age 2	Sensorineural
WU.WOLF-07	Normosmia	High Average	Loss of pituitary bright spot	Yes, on right only—diagnosed at clinic	Conductive
WU.WOLF-09	Mild Microsmia	High Average	Elevated T2 in the pons	No	Normal
WU.WOLF-10	Normosmia	Average	Several areas of T2 elevation	No	Normal
WU.WOLF-11	Total Anosmia	High Average	Normal	Yes—diagnosed at clinic	Mixed
WU.WOLF-12	Total Anosmia	Average	Several small T2 anomalies	Yes—hearing aid at age 7	Sensorineural
WU.WOLF-13	Total Anosmia	Average	Small posterior pituitary	Yes—diagnosed at clinic; no hearing aids	Sensorineural
WU.WOLF-14	Normosmia	High Average	Elevated T2 in the periventricular occipital lobe	Yes—hearing aids	Sensorineural
WU.WOLF-15	Total Anosmia	Superior	Faint pituitary bright spot	Yes—No hearing aids	Sensorineural
WOLF2011-16	Moderate Microsmia	High Average	Multiple T2 abnormalities, punctate in all lobes. Poorly seen bright pituitary focus, either small or absent.	Yes—No hearing aids	Sensorineural
WU.WOLF-17	Mild Microsmia	High Average	Multiple punctate T2 foci in frontal lobes. Pituitary bright spot not seen	Yes—No hearing aids	Sensorineural
WU.WOLF-18	Mild Microsmia	Very Superior	Absent pituitary bright spot	Yes—hearing aids	Sensorineural
WU.WOLF-19	Mild Microsmia	ND	ND	Yes—No hearing aids	Sensorineural
WU.WOLF-22	Normosmia	Average	Several small foci of elevated flair signal	Yes—Cochlear Implants	Sensorineural

ND = testing not done.

Verbal IQ was overall very high, with 15 of the 16 tested performing at the average range or above. The subject with a history of near drowning performed in the borderline range.

### Clinical neuroimaging findings

Three subjects could not undergo MRI neuroimaging due to medical implants and or anxiety. Thus, scans were obtained on 14 subjects. Pituitary abnormalities (reduced or absent bright spot on T1-weighted images or small posterior pituitary) were seen in seven (50%) of the scans. Of these seven, five had diabetes insipidus, but 6 (67%) of the subjects who were scanned who had diagnosed diabetes insipidus did not have an abnormal pituitary. In addition, T2-weighted or flair imaging showed signal abnormalities in pons (one, 7%) or cortex (five, 36%).

No other clinically detectable abnormalities were noted. However, reduced volume of the brainstem and cerebellum have been found compared to age-matched controls (data previously published [26]).

### Vision assessment

Pallor of the optic disc was present in 17 subjects (94%). The one subject with a normal optic disc, interestingly, did have a color vision defect, acuity defect and a cataract (Table 6). Abnormalities in visual acuity and color vision were present in 17 (94%). Abnormal pupillary response, nystagmus, and cataracts were present in 11 (61%), five (28%), and five (28%), respectively. Interestingly, six (33%) of the subjects noted loss of their color vision around age 6, most long before any vision problems were formally diagnosed. In five (27%) of the subjects, school screening or teachers were the first to identify a vision problem, which led in some cases to the diagnosis of WFS. The finding of optic atrophy led to the diagnosis of WFS in seven (39%), but three (17%) were diagnosed with optic atrophy for 2 to 10 years before the diagnosis of WFS was made.

**Table 6:** Vision findings

**Subject**	**Visual acuity (Eyes together)**	**Abnormal pupillary response**	**Color vision defect**	**Nystagmus**	**Cataracts**	**Optic disc pallor**
WU.WOLF-01	20/60	Yes	Strong defect	Yes	No	Yes
WU.WOLF-02	20/50	Yes	Strong defect	No	No	Yes
WU.WOLF-03	20/200	Yes	Strong defect	No	Yes: Peripheral spokes	Yes
WU.WOLF-04	Hand motion only	Yes	Strong defect	Yes	No	Yes
WU.WOLF-05	20/70	No	Moderate defect	No	Yes: Snowflake	Yes
WU.WOLF-07	20/20	No	No	No	No	Trace
WU.WOLF-09	20/250	Yes	Strong defect	No	No	Yes
WU.WOLF-10	20/250	Yes	Strong defect	No	No	Yes
WU.WOLF-11	20/125	Yes	Strong red/green defect	Yes	No	Yes
WU.WOLF-12	20/80	Yes	Strong defect	Yes	Yes: Lamellar	Yes
WU.WOLF-13	20/60	No	Moderate defect	No	No	Yes
WU.WOLF-14	20/250	Yes	Strong defect	No	No	Yes
WU.WOLF-15	20/60	Yes	Strong defect	No	No	Yes
WU.WOLF-16	20/100	No	Strong defect	No	Yes: Snowflake	Yes
WU.WOLF-17	20/25	No	Mild red/green defect	No	No	Yes
WU.WOLF-18	20/40	Yes	Strong red/green defect	Yes	No	Yes
WU.WOLF-19	20/50	No	Moderate red/green defect	No	Yes: Posterior subcapsular	No
WU.WOLF-22	20/40	No	Strong defect	No	No	Yes

### Audiology findings

Normal hearing was found in four subjects (22%). Profound hearing loss with onset before age 2 was present in one and one other probably also had very early profound hearing loss as noted above.

With respect to vestibular function, one subject had reduced gain of the vestibulo-ocular reflex to steps of angular acceleration and reduced gain and increased low-frequency phase lead on sinusoidal harmonic testing over the range 0.025–0.5 Hz. This provides for the first time quantitative evidence that WS can cause profound vestibular loss in a subset of subjects.

### Urology findings

One subject had chronic renal failure and neurogenic bladder diagnosed at age 17. Enuresis was reported by four (22%) and nocturia by three (17%). Of the 11 tested for bladder emptying, five (45%) had elevated post-void residual bladder volume.

## Discussion

WFS is a rare monogenic disorder characterized by ER stress in cell lines [4,6,27] and in animal models [4,5,7] with multiple clinical manifestations. Comprehensive, standardized phenotyping of subjects in the early stages of this syndrome is limited. This study identified many clinically detectable symptoms present in a group of individuals with WFS in its relatively early stages. These data contribute to our understanding of the natural history of the clinical features of this complex syndrome, which could facilitate diagnosis with WFS and ultimately be useful in future therapeutic trials.

In many subjects a significant delay occurred between the time of the diagnosis of the first signs of the syndrome and the diagnosis of WFS. In some cases the delay led to prolonged periods of polyuria, vision problems, or hearing problems before those features were diagnosed and treated. Primary care physicians, ophthalmologists, and endocrinologists were all likely to be the caregivers first proposing the diagnosis and school screening programs were instrumental in identifying vision and hearing losses in several. Earlier diagnosis may improve the quality of life of subjects with WFS by leading to earlier diagnosis and intervention of all of the features of WFS. Diabetes insipidus and bladder issues in particular are difficult to diagnose in a subject with pre-existing diabetes mellitus given that polyuria and polydipsia are features of both. It is likely that there are subjects who might be diagnosed even before the onset of diabetes mellitus or insipidus if there is an index of suspicion for the disorder in subjects with hearing loss, color vision defects, gait abnormalities, etc. We suggest that the addition of certain questions on review of systems in subjects with diabetes, in particular, could lead to earlier diagnosis of the syndrome. For example, color blindness and loss of olfaction are common, even in the youngest subjects with WFS, and were frequently noticed by the subjects but not mentioned to caregivers. Both can be rather easily and inexpensively screened in an outpatient primary care or endocrinology clinic setting in the event a patient reports symptoms.

The literature indicates that WFS is a progressive neurodegenerative disorder with severe neurologic abnormalities and often death during the third or fourth decade of life. However, the order in which symptoms emerge over time in WFS has been difficult to discern. A recent paper surveying (but not directly assessing) 59 subjects with WFS ranging in age from 5 to 54 found that some individuals are reported to have optic atrophy prior to the diagnosis of diabetes mellitus [17]. These investigators did not test each subject directly, and did not assess olfaction or color vision, so it is not clear whether these features were also present prior to diabetes. Our study indicates that individuals with WFS may manifest multiple abnormalities of the sensory (vision, hearing and olfactory), neurologic and endocrine systems relatively early in the disease process. In particular, our limited sample suggests that optic atrophy may not always predate other clinically apparent neurologic findings (i.e. WU.WOLF-19). However, this study is limited by the small number of subjects evaluated and may have a bias toward higher functioning families with the interest and means to attend the clinic. Future clinics will include a larger number of subjects, but international cooperation and standard assessment tools, such as a rating scale for severity (e.g. the Wolfram Unified Rating Scale or WURS [28]) will be necessary to fully address this issue. Whether the additional manifestations we identified predict the long-term course of WFS will require longitudinal follow-up of this and other cohorts. Since the majority of subjects with WFS have diabetes mellitus at the time that WFS is diagnosed, these other sensory and neurological manifestations may be important for following the natural history of WFS and testing the effect of potential interventions.

The WFS1 gene is one of the genes repeatedly shown to be associated with Type 2 diabetes [29-32], indicating that WFS may be an unusual example of a Type 2-like diabetes mellitus of monogenic cause. This characteristic may present an opportunity for testing diabetes therapies in a monogenic setting or further delineating the mechanism of disease in Type 2 diabetes. The WFS1 gene encodes for the Wolframin protein that is an ER membrane protein recently shown to control the degree of ER stress [4]. A number of neurodegenerative disorders including Alzheimer’s, Huntington’s, and prion diseases have also been proposed as disorders of the ER stress response, suggesting that WFS may also represent a useful model of much more common neurodegenerative diseases [33]. It is encouraging that therapeutic agents to treat ER-stress-related diseases are actively being developed [34].

WFS causes multiple endocrine and neurologic deficits, even early in the course of the disease that are clinically apparent when subjects are directly examined in a standardized manner. The sensory system appears particularly affected, with defects found in vision, hearing, the vestibular system, and olfaction soon after diagnosis with diabetes. Understanding the evolution of these symptoms over time in WFS is a necessary step towards preparing for future clinical trials.

## Abbreviations

WFS: Wolfram syndrome; ER: Endoplasmic reticulum; MRI: Magnetic resonance imaging; WASI: Wechsler abbreviated scale of intelligence; UPSIT: University of Pennsylvania smell identification test; IQ: Intelligence quotient

## Competing interests

The authors report no conflicts of interest.

## Authors’ contributions

BM serves as medical director of the research clinic and, together this NHW designed and carried out the questionnaires and examinations relating to the subjects endocrine and general medical issues. BM was also responsible for drafting the article and final writing of the version to be published. MAP conceived the project and made substantial contributions to design of the clinic, identification of appropriate subjects and recruitment. AP, JH, RK, AV, LM, PA, TEH, and TH made substantial contributions to conception and design, acquisition of data, and analysis and interpretation of data in the domains of neurology, vision, hearing, neurology, genetics, urology, otolaryngology, and psychiatry, respectively. JW made substantial contributions to conception and design and subject selection and recruitment. JS and TH carried out and interpreted the radiological studies. All of the authors participated in analysis and interpretation of data, revising the article critically for important intellectual content; and final approval of the version to be published (with the exception of MAP who sadly passed away prior to submission of the final version of the manuscript).

## Authors’ information

Washington University Wolfram Study Group Members:

Paul Austin, M.D. (Surgery)

Aiden Bondurant, B.S. (Psychiatry)

Gammon Earhart, Ph.D. (Physical Therapy)

Tamara Hershey (Psychiatry, Neurology, Radiology)

James Hoekel, O.D. (Ophthalmology and Visual Sciences)

Timothy Hullar, M.D. (Otolaryngology)

Roanne Karzon, Ph.D. (Audiology & Communication Sciences, St. Louis Children’s Hospital)

Heather M. Lugar, M.A. (Psychiatry)

Linda Manwaring, M.S. (Pediatrics)

Bess Marshall M.D. (Pediatrics)

Chau Nguyen, B.S. (Occupational Therapy)

Alex R. Paciorkowski, M.D. (Neurology, U Rochester)

M. Alan Permutt, M.D. (Internal Medicine)

Kristen Pickett, Ph.D. (Physical Therapy)

Jerrel Rutlin, B.A. (Psychiatry)

Joshua Shimony, M.D., Ph.D (Radiology)

Amy Viehoever, M.D. (Neurology)

Jon Wasson B.S. (Internal Medicine)

Neil H. White M.D., CDE (Pediatrics)

Fumihiko Urano, M.D., Ph.D. (Internal Medicine)

Note: all are located in the departments noted at Washington University School of Medicine, St. Louis, MO except Dr. Paciorkowski, who is located at the University of Rochester, Rochester, NY.
